# Supplementary material for: Pseudomonas sp. Strain ADAl3–4 Enhances Aluminum Tolerance in Alfalfa (Medicago sativa)
Source: Int J Mol Sci. 2025 May 20;26(10):4919. doi: 10.3390/ijms26104919 (PMC12111897; doi:10.3390/ijms26104919)
Supplement: Supplementary file 1 [file ijms-26-04919-s001.zip › Supplementary FigureS1-S4.pdf]

Supplementary Figure S1- S4

Figure S1 Strain ADAl3–4 promoted plant growth. (A) Phenotypic responses of 10-d-old A. thaliana seedlings under aluminum stress (450 uM Al^{3+}) with/without application if strain ADAl3-4. Scale bar = 1 cm. (B) Statistics of root length in (A). n = 30, three independent experiments per sample.

Figure S2 Strain ADAl3-4 promoted maize growth. (A) Phenotypic analysis of one-month-old maize seedlings under 1,100 mg/kg Al treatment. (B) Statistics of maize seedling biomass in A. (C) Statistics of antioxidant enzyme activities. (D-F) Statistics of contents of MDA, proline and chlorophyll. Three biological replicates per sample. *, $P < 0.05$; **, $P < 0.01$; ***, $P < 0.001$; ns, no significance. Student's t-test.

Figure S3 DEGs with opposite trends among H2O-CK, Al-CK and Al+ADAl3-4. novel genes were excluded.

Figure S4 GO enrichment analysis. (A, B) GO enrichment categories of the indicated groups. (C-E) Gene Ontology (GO) enrichment of DEGs in Biological processes (C), Molecular functions (D), and Cellular components (E) for Al-CK_vs_H2O-CK comparison. The color intensity reflects relative significance: red (most significant, $P < 0.0001$), orange ($P < 0.001$), light yellow ($P < 0.05$), and white (non-significant).

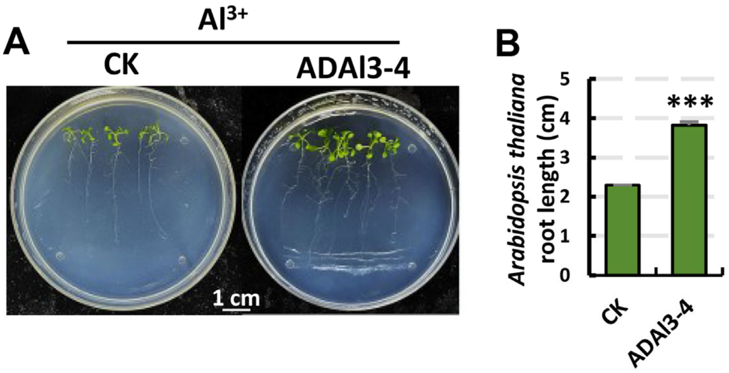

Figure S1

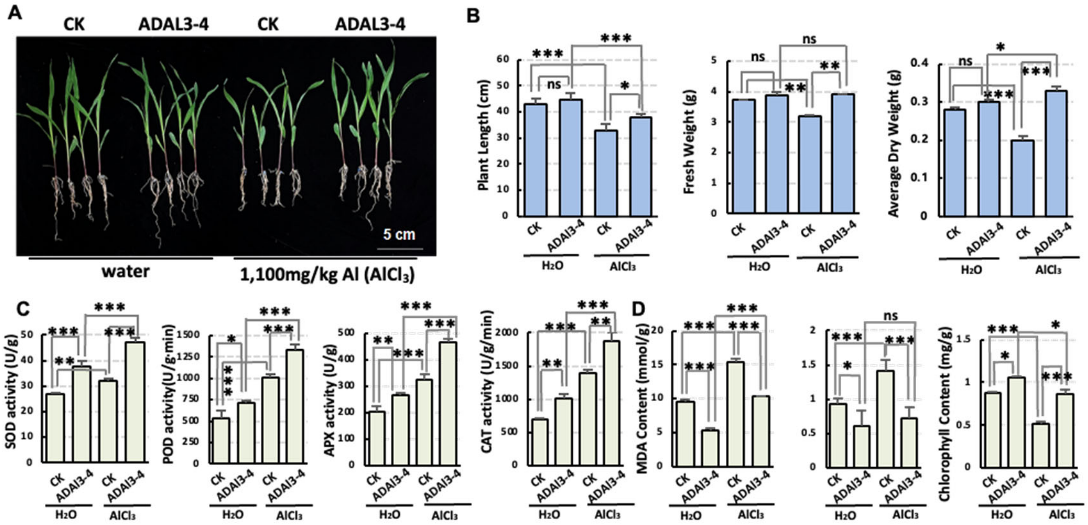

Figure S2

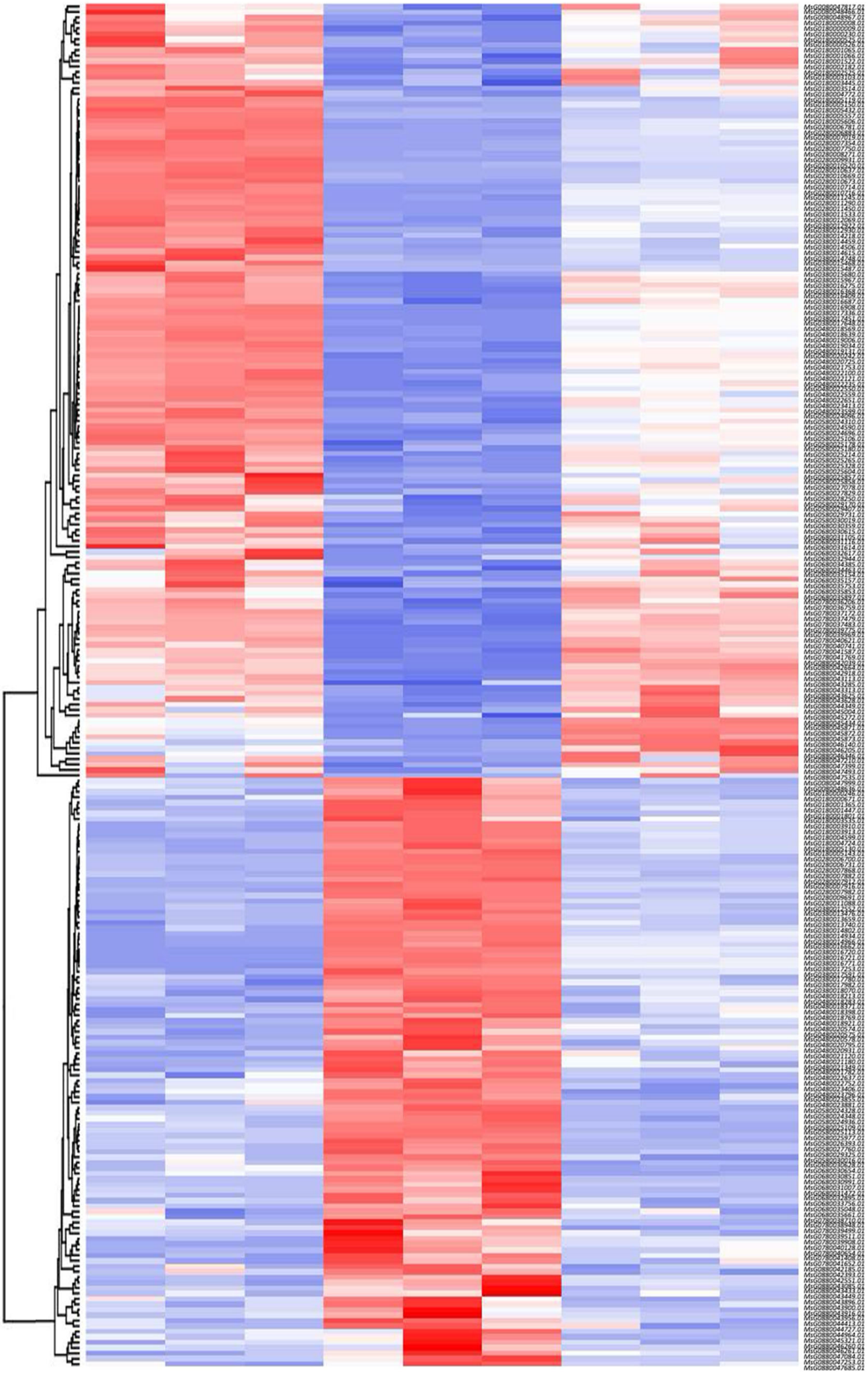

Figure S3

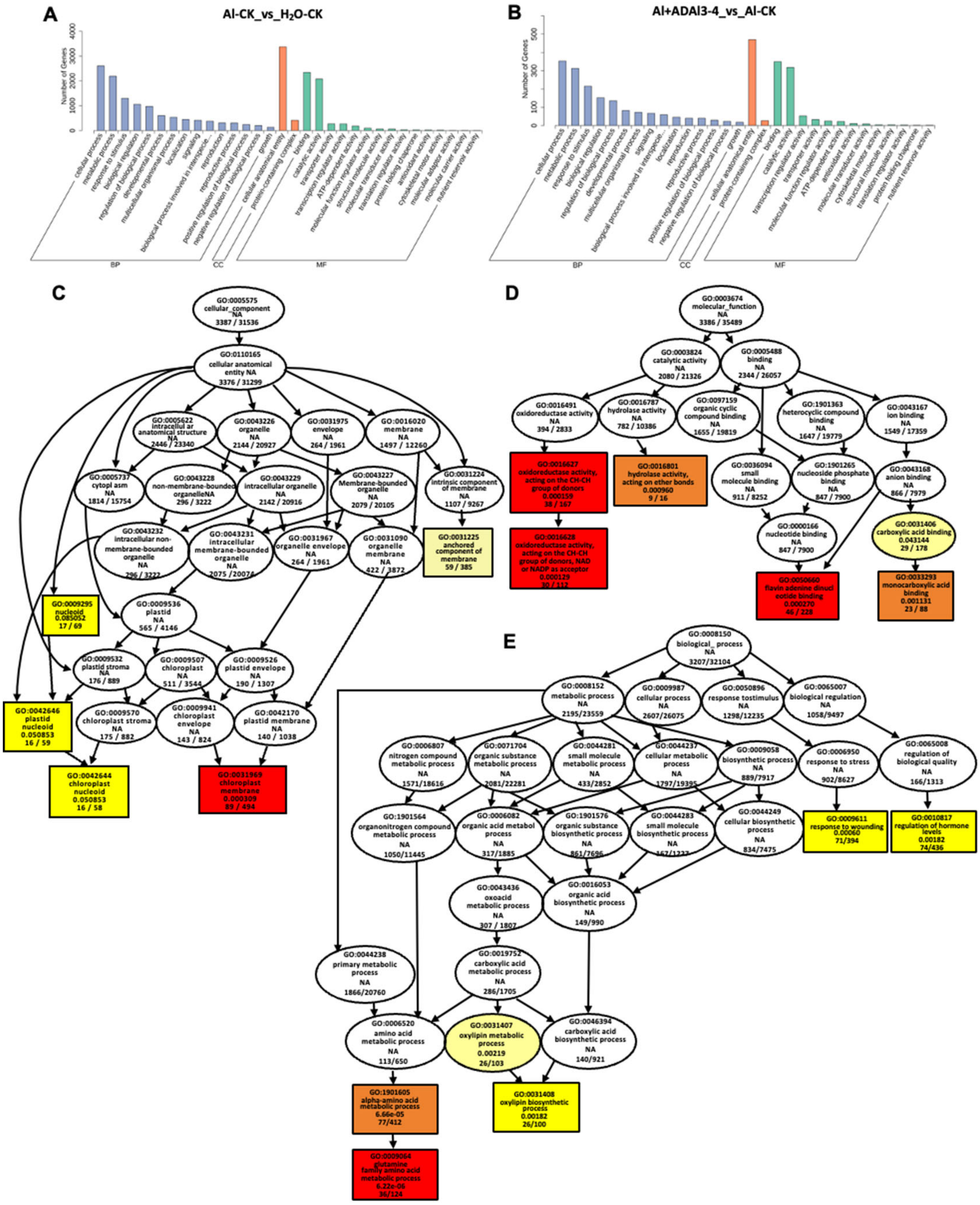
A
Al-CK_vs_H$_2$O-CK
B
Al+ADAl3-4_vs_Al-CK
Number of Genes
BP
CC
MF
C
GO:0005575 cellular_component NA 3387 / 31536
GO:0110165 cellular anatomical entity NA 3376 / 31299
GO:0005622 intracellular anatomical structure NA 2446 / 23340
GO:0043226 organelle NA 2144 / 20927
GO:0031975 envelope NA 264 / 1961
GO:0016020 membrane NA 1497 / 12260
GO:0005737 cytoplasm NA 1814 / 15754
GO:0043228 non-membrane-bounded organelle NA 296 / 3222
GO:0043229 intracellular organelle NA 2142 / 20916
GO:0043227 Membrane-bounded organelle NA 2079 / 20105
GO:0031224 intrinsic component of membrane NA 1107 / 9267
GO:0043232 intracellular non-membrane-bounded organelle NA 296 / 3222
GO:0043231 intracellular membrane-bounded organelle NA 2075 / 20074
GO:0031967 organelle envelope NA 264 / 1961
GO:0031090 organelle membrane NA 422 / 3872
GO:0031225 anchored component of membrane 59 / 385
GO:0009295 nucleoid 0.085052 17 / 69
GO:0009536 plastid NA 565 / 4146
GO:0009532 plastid stroma NA 176 / 889
GO:0009507 chloroplast NA 511 / 3544
GO:0009526 plastid envelope NA 190 / 1307
GO:0042646 plastid nucleoid 0.050853 16 / 59
GO:0009570 chloroplast stroma NA 175 / 882
GO:0009941 chloroplast envelope NA 143 / 824
GO:0042170 plastid membrane NA 140 / 1038
GO:0042644 chloroplast nucleoid 0.050853 16 / 58
GO:0031969 chloroplast membrane 0.000309 89 / 494
D
GO:0003674 molecular_function NA 3386 / 35489
GO:0003824 catalytic activity NA 2080 / 21326
GO:0005488 binding NA 2344 / 26057
GO:0016491 oxidoreductase activity NA 394 / 2833
GO:0016787 hydrolase activity NA 782 / 10386
GO:0097159 organic cyclic compound binding NA 1655 / 19819
GO:1901363 heterocyclic compound binding NA 1647 / 19779
GO:0043167 ion binding NA 1549 / 17359
GO:0016627 oxidoreductase activity, acting on the CH-CH group of donors 0.000159 38 / 167
GO:0016801 hydrolase activity, acting on ether bonds 0.000960 9 / 16
GO:0036094 small molecule binding NA 911 / 8252
GO:1901265 nucleoside phosphate binding NA 847 / 7900
GO:0043168 anion binding NA 866 / 7979
GO:0016628 oxidoreductase activity, acting on the CH-CH group of donors, NAD or NADP as acceptor 0.000129 30 / 112
GO:0000166 nucleotide binding NA 847 / 7900
GO:0031406 carboxylic acid binding 0.043144 29 / 178
GO:0050660 flavin adenine dinucleotide binding 0.000270 46 / 228
GO:0033293 monocarboxylic acid binding 0.001131 23 / 88
E
GO:0008150 biological_process NA 3207/32104
GO:0008152 metabolic process NA 2195/23559
GO:0009987 cellular process NA 2607/26075
GO:0050896 response to stimulus NA 1298/12235
GO:0065007 biological regulation NA 1058/9497
GO:0006807 nitrogen compound metabolic process NA 1571/18616
GO:0071704 organic substance metabolic process NA 2081/22281
GO:0044281 small molecule metabolic process NA 433/2852
GO:0044237 cellular metabolic process NA 1797/19395
GO:0009058 biosynthetic process NA 889/7917
GO:0006950 response to stress NA 902/8627
GO:0065008 regulation of biological quality NA 166/1313
GO:1901564 organonitrogen compound metabolic process NA 1050/11445
GO:0006082 organic acid metabolic process NA 317/1885
GO:1901576 organic substance biosynthetic process NA 861/7696
GO:0044283 small molecule biosynthetic process NA 167/1227
GO:0044249 cellular biosynthetic process NA 834/7475
GO:0009611 response to wounding 0.00060 71/394
GO:0010817 regulation of hormone levels 0.00182 74/436
GO:0043436 oxoacid metabolic process NA 307/1807
GO:0016053 organic acid biosynthetic process NA 149/990
GO:0044238 primary metabolic process NA 1866/20760
GO:0019752 carboxylic acid metabolic process NA 286/1705
GO:0006520 amino acid metabolic process NA 113/650
GO:0031407 oxylipin metabolic process 0.00219 26/103
GO:0046394 carboxylic acid biosynthetic process NA 140/921
GO:1901605 alpha-amino acid metabolic process 6.66e-05 77/412
GO:0031408 oxylipin biosynthetic process 0.00182 26/100
GO:0009064 glutamine family amino acid metabolic process 6.22e-06 36/124

Figure S4
